# Self-care adherence and affective disorders in Barbadian adults with type 2 diabetes

**DOI:** 10.3934/publichealth.2022006

**Published:** 2021-11-17

**Authors:** Allison DaSantos, Carlisle Goddard, Dalip Ragoobirsingh

**Affiliations:** 1 University of the West Indies, Faculty of Medical Sciences, Cave Hill Campus Cave Hill Bridgetown, Barbados; 2 University of the West Indies, Faculty of Medical Sciences, Bridgetown, Barbados; 3 Professor-Medical Biochemistry and Diabetology, Director-UWI Diabetes Education Programme, University of the West Indies Faculty of Medical Sciences Teaching & Research Complex (Level 2) Mona, Kingston 7, Jamaica

**Keywords:** Barbados, adherence, diabetes distress, depression, type 2 diabetes

## Abstract

**Purpose:**

Diabetes management requires adherence to complicated self-care behaviors. Therefore, the emotional state of the individual living with diabetes, is likely to affect their ability to comply with recommendations. This study explored the relationship of self-care adherence to diabetes distress and depression in Barbadian adults with type 2 diabetes.

**Methods:**

Adults aged 20 to 80 years, with type 2 diabetes, completed self-report questionnaires comprised of a profile section consisting of demographic and clinical characteristics; and standardized questionnaires comprising, The Diabetes Distress Scale (DDS), The Patient Health Questionnaire (PQH-9), and the Summary of Diabetes Self-care Activities Scale (SDSCA). Additionally biological measures (BP and HbA1c) were collected.

**Results:**

For the 509 participants there were no differences in adherence for males (30.8%) and females (69.2%), high diabetes distress and depression were associated with low adherence. General diet was negatively associated with BP and HbA1c; while HbA1c was positively correlated with blood glucose testing.

**Conclusion:**

Self-care non-adherence is more than a behavioral problem; it is a multidimensional phenomenon inclusive of demographic factors, condition or disease factors, psychological and social factors.

## 1. Introduction

Poor adherence to therapies gravely jeopardizes the efficacy of treatment, yet, many patients experience difficulty following treatment, making this a major problem in public health [1]. And, despite the ubiquitous nature of the issue it continues to be a challenge for both social scientists and medical professionals [2] with an estimated 20% to 50% of patients considered to be non-adherent to medical therapy [3]. Seemingly, the group of individuals most affected by this phenomenon is those affected by chronic illness.

Chronically ill patients have to deal with the loss of independence, the threat of disease advancement and in many cases, the demands of changing their behaviors to meet the challenges of the recommended treatment regimen. Progressively, the particular medical treatments can be self-administered but inadequate, or non-adherence can compromise the effectiveness of treatment [4]. Unfortunately, few patients choose to make the necessary changes [5]. As a result, the complexity and importance of non-adherence remains a challenging issue for clinicians and patients alike; reducing quality of life and placing an economic strain on the society.

Due to the high prevalence of the chronic illness diabetes in the Caribbean (approximately 13.1%) [6], the region is undoubtedly not immune to the occurrence of non-adherence. Quantitative studies conducted within the region offer some insight on levels of adherence to diabetic recommendations. In Trinidad, for example, Del Pino and colleagues (2015), in determining the extent of adherence to disease management strategies, found that over 50% of diabetic patients were not well controlled [7]. Additionally, a cross-sectional study found that three times as many women than men were compliant in clinic attendance and, women were more likely to be compliant with dietary regimens and taking medication [8]. And, in Jamaica, an assessment of self-care practices and their relationship to glycemic control, reported that only 45% of patients were fully compliant with taking medication. Moreover, while 85% consulted a dietician, only 56.4% reported being on a ‘special diet’. It was also found that HbA1c and BMI increased with less self-care and a third of persons reported not exercising at all [9].

While achieving beneficial therapeutic results requires adequate and appropriate adherence to recommended regimens, there are numerous issues that may be partly responsible for whether a patient with diabetes adheres to the recommended treatment [10]. An important aspect to addressing the issue of non-adherence therefore, is the acquisition of an understanding of the multiple factors that interact to determine the efficacy of the prescribed treatment. Although some persons who face the continued stress of dealing with a chronic disease become well adjusted, others in contrast express a significant emotional and personal downturn. Undeniably, the experience of having to live with chronic illness such as diabetes can cause some lifestyle disruption and, bring about psychological consequences. A biopsychosocial model acknowledges that social and psychological factors impact a patient's perceptions and actions and thus the reality of feeling ill [11],[12]. Because of the appreciation that the biological, social and psychological levels encourage the recognition and understanding that cessation and maintenance are the result of various processes in combination over time [13],[14], it is essential to capture the complexity of the processes which contribute to illness prevention and treatment [15].

Research has clearly demonstrated that there is an association between the psychological factors of depression in patients with diabetes. However, while depression has been shown to affect diabetes treatment outcome and self-management behavior, existing evidence also points to diabetes distress (the emotional state in which persons with diabetes experience feelings of guilt, denial and stress as a result of having to live with and manage their illness [16]); as a major factor associated with treatment outcomes and mental well-being of diabetic patients with diabetes. Notwithstanding the existence of research literature on this aspect of diabetic treatment, there is in the Caribbean, a paucity of research which has been conducted in this area. The Caribbean research on diabetes has primarily focused on epidemiological statistics such as prevalence and incidence of diabetes, its risks factors and its complications [17]–[19]. There is yet no literature that supports that there is a problem with psychological disorders such as diabetes distress and depression in persons with diabetes in Barbados; however, that the outcomes for diabetes is less than optimal [20], may be indicative that these variables may be associated with outcomes, as studies in other countries have demonstrated the probable relationships.

The heavy psychological toll of living with diabetes can often affect self-care behavior and eventually, lead to long-term complications and evidence suggests that psychological issues are crucial to diabetes care [21]. Moreover, psychological factors frequently influence self-management behaviors, and psychological variables like depression and diabetes distress can often be greater predictors than physiologic measures, in the prediction of such outcomes as mortality and hospitalizations [22]. Meanwhile, the presence of psychological issues can often result in poor clinical outcomes because of its possible effect on adherence to medication and self-care regimes [23]. As diabetes management is largely dependent upon patients' mental state which may affect their ability to adhere to a complex set of self-care behaviors; the aim of this study was to explore for the first time as far as we are aware, the relationship of self-care adherence, and the affective emotional disorders of diabetes distress (DD) and depression among Barbadian adults with type 2 diabetes.

## 2. Materials and methods

This study was undertaken, employing a cross-sectional survey which sought to describe the frequency of the particular attribute (diabetes distress, depression, and adherence) within the defined population (persons with type 2 diabetes). The aim of this survey was to gather information about the present and past occurrences, and to investigate the associations between the variables [24].

The study was conducted in Barbados. Permission was sought and acquired from the Institutional Research Board and Ministry of Health of Barbados, for the investigator to conduct research within the public polyclinics throughout the island. The inclusion criteria were: persons diagnosed with type 2 diabetes between the ages of 20 and 80, the exclusion criteria was an absence of severe diabetes complications or functional deficits (dialysis, blindness), and no diagnosis of psychosis or dementia.

Persons attending the polyclinics are usually seen by a nurse(s) prior to their consultation with the physician. These nurses are therefore able to identify from their charts the patients who are diagnosed with type 2 diabetes. The nurse(s) then informed the investigator, who then systematically randomized those patients to approach and requested their participation in the study. All participants were assured that their participation was completely discretionary and that if they choose, they may withdraw at any time. All who agreed to participate signed a consent form. For the collection of quantitative data, a questionnaire battery (paper-pencil form) was utilized to obtain participants' self-reports.

The questionnaire comprised of two parts. Part One: encompassed questions on socio-demographic characteristics such as: sex, age, marital status; as well as questions on clinical characteristics such as: presence of co-morbid disease and disease related complications, and duration of illness. Part Two: consisted of: The Diabetes Distress Scale (DDS), which sought to screen for and measure the level of diabetes distress [25]; (In this study the Cronbach alpha coefficient was 0.94) The Patient Health Questionnaire (PQH-9), which sought to screen for and measure the severity of depression. [26] in this study the Cronbach alpha coefficient was 0.82 and the Summary of Diabetes Self-care Activities Scale (SDSCA), which sought to measure levels of self-management across different components of the diabetes regimen [27]. The areas of regimen assessed were diet, exercise, glucose testing and foot-care. The SDSCA is an 11-item self-report questionnaire. Scores are calculated for each area creating five subscales measured by the SDSCA: diet, exercise, blood glucose testing, foot checks. Arithmetical scores of items are based upon the number of days of the week that the behavior was performed (0–7). A mean number is then computed for each part by obtaining the numerical mean of the standard scores. Six additional items are offered in the SDSCA which would allow for evaluation of congruence between perceived recommendations and levels of adherence reported. However, given that the initial items of the SDSCA provides adequate information for the scope of the current study, and because the additional items would incur more time to complete, impinging on the time resource, they were excluded. The scores for the Summary of Diabetes Self-care Activities Scales (SDSCA) were calculated as follows: For items 1–10, the number of days per week were used on a scale of 0–7. General Diet — Mean number of days for items 1 and 2. Specific Diet — Mean number of days for items 3, and 4, reversing item 4 (0 = 7, 1 = 6, 2 = 5, 3 = 4, 4 = 3, 5 = 2, 6 = 1, 7 = 0). Exercise — Mean number of days for items 5 and 6. Blood-Glucose Testing — Mean number of days for items 7 and 8. Foot-Care — Mean number of days for items 9 and 10 [27]. In this study the Cronbach alpha coefficient was 0.70. Cronbach alpha is reported as a value between 0 and 1 and so the decimal point is required.

In addition, biological (HbA1c and blood pressure) measures were collected.

The population of this study consisted of 509 persons diagnosed with type 2 diabetes and registered at the public polyclinics on the island. Although 572 persons where approached, only 555 individuals fit the criteria for eligibility, of these, 511 persons agreed to participate, with 509 providing viable data. Quantitative data collected were then analyzed using SPSS (Statistical Packages for Social Sciences) version 24.0 for Windows software. Each statistical test was two-sided and a p-value of ≤0.05 was deemed statistically.

## 3. Results

**Table 1.** Descriptive statistics by gender for measures of adherence.

Gender		SDSCA Gender Diet	SDSCA Specific Diet	SDSCA Exercise	SDSCA Blood Glucose Testing	SDSCA Foot Care
Male	N	157.00	157.00	157.00	157.00	157.00
		0.00	0.00	0.00	0.00	0.00
	Mean	4.45	4.32	3.39	3.05	4.20
	Median	5.00	4.00	3.50	2.00	3.50
	Std. Deviation	2.24	1.72	2.37	2.62	2.46
Female	N	352.00	352.00	352.00	352.00	352.00
		0.00	0.00	0.00	0.00	0.00
	Mean	4.37	4.63	3.20	3.22	4.31
	Median	4.50	5.00	3.50	3.00	3.75
	Std. Deviation	2.11	1.61	2.08	2.63	2.38

The overall sample in the dataset for analysis consisted of 509 participants, 30.8% males (n = 157) and 69.2% females (n = 352) with an overall mean age of 63.54 (SD = 11.73). Table 1 presents the descriptive statistics by gender for adherence.

Spearman's Rho. revealed strong negative relationships between all the measures of SDSCA and diabetes distress, with high levels of diabetes distress associated with lower levels of adherence. (Table 2).

**Table 2.** Spearman's Rho correlations between measures of diabetes self-care adherence and diabetes distress.

Scale	Total Diabetes Distress Scores	SDSCA General Diet	SDSCA Specific Diet	SDSCA Exercise	SDSCA Blood Glucose Testing	SDSCA Foot Care
Total Diabetes Distress Scores	-	-	-	-	-	-
General Diet	−0.28**	-	-	-	-	-
Specific Diet	−0.17**	0.34**	-	-	-	-
Exercise	−0.20**	0.13**	0.19**	-	-	-
Blood Glucose Testing	−0.11*	0.11*	0.14**	0.05	-	-
Foot Care	−0.22**	0.25**	0.21**	0.32**	0.08	-

*Note: ** Correlation is significant at the 0.01 level (2-tailed); * Correlation is significant at the 0.05 level (2-tailed).

Spearman's Rho. revealed strong negative relationships between all the measures of SDSCA and Depression, with high levels of depression associated with low levels of adherence. See Table 3.

**Table 3.** Spearman's Rho correlations between measures of diabetes self-care adherence and depression.

Scale	Total Patient Health Questionnaire	SDSCA General Diet	SDSCA Specific Diet	SDSCA Exercise	SDSCA Blood Glucose Testing	SDSCA Foot Care
SDSCA General Diet	−0.18	-	-	-	-	-
SDSCA Specific Diet	−0.15**	0.34**	-	-	-	-
SDSCA Exercise	−0.21**	0.13**	0.19**	-	-	-
SDSCA Blood Glucose Testing	−0.02	0.11*	0.14**	0.05	-	-
SDSCA Foot Care	−0.21**	0.25**	0.20**	0.32**	0.08	-
Depression (PHQ-9)	-	−0.18**	−0.15**	−0.21**	−0.01	−0.21**

*Note: ** Correlation is significant at the 0.01 level (2-tailed); * Correlation is significant at the 0.05 level (2-tailed).

The relationships between measures of diabetes self-care adherence and the biological measures (systolic blood pressure (BP), diastolic blood pressure and glycated hemoglobin) (A1c) were investigated using Spearman's Rho. There were negative correlations between all three clinical measures and general diet, while there was a positive correlation between glycated hemoglobin and sugar testing. Table 4 shows the correlations between the biological measures and the measures of self-care adherence. See Table 4.

**Table 4.** Spearman's Rho correlations between measures of adherence, systolic BP, diastolic BP and A1C.

	Correlations							
	Systolic Blood Pressure (mm/Hg)	Diastolic Blood Pressure (mm/Hg)	Glycated Haemoglobin level(A1c)	SDSCA General Diet	SDSCA Specific Diet	SDSCA Exercise	SDSCA Blood Glucose Testing	SDSCA Foot Care
Systolic Blood Pressure (mm/Hg)	-	-	-	−0.03	0.09	0.06	−0.02	0.10*
Diastolic Blood Pressure (mm/Hg)	0.55**	-	-	−0.10*	−0.00	−0.04	−0.10*	−0.02
Glycated Haemoglobin level(A1c)	0.17**	0.15**	-	−0.12**	−0.07	−0.08	0.12**	−0.05

*Note: ** Correlation is significant at the 0.01 level (2-tailed); * Correlation is significant at the 0.05 level (2-tailed).

The relationships between measures of diabetes self-care adherence and age were investigated using Spearman's Rho. There was a significant positive correlation between age and general diet, as well as age and foot-care, Table 5 shows the correlation coefficients for measures of diabetes self-care adherence and age.

**Table 5.** Spearman's Rho correlations between measures of diabetes self-care adherence and age.

	Age	SDSCA General Diet	SDSCA Specific Diet	SDSCA Exercise	SDSCA Blood Glucose Testing	SDSCA Foot Care
General Diet	0.19**	-	-	-	-	-
Specific Diet	0.07	0.34**	-	-	-	-
Exercise	0.06	0.13**	0.19**	-	-	-
Blood Glucose Testing	0.07	0.11*	0.14**	0.05	-	-
Foot Care	0.10*	0.25**	0.20**	0.32**	0.08	-
Age	-	0.19**	0.07	0.06	0.07	0.10*

*Note: ** Correlation is significant at the 0.01 level (2-tailed); * Correlation is significant at the 0.05 level (2-tailed).

Independent samples T-tests were conducted to compare the diabetes distress scores for participants scores on the five measures of diabetes self-care adherence. There were no statistically significant differences in any of the mean adherence scores for male and female participants.

Direct Logistic Regression was performed to assess and identify the impact of factors on the likelihood that respondents would have Depression. The model contained five independent variables (Days of Adherence to: General Diet, Specific diet, Exercise, blood glucose testing and Foot-care). The full model containing all predictors was statistically significant, χ^2^ (5, N = 509) = 34.84, p < 0.001, indicating that the model was able to distinguish between respondents who had and did not have depression. The model as a whole explained between 6.6% (Cox and Snell R square) and 9.1% (Nagelkerke R squared) of the variance in depression status, and correctly classified 66.8% of cases. Only two of the independent variables made a unique statistically significant contribution to the model (Adherence to: Exercise and Foot-care). The strongest predictor of reporting depression was Exercise, recording an odds ratio of 0.88 This indicated that the more days that a person adheres to Exercise regime, the odds of him or her having depression decreases by a factor of 88, controlling for all other factors in the model. The odds ratio of 0.87 for Adherence to Foot-care was also less than 1, indicating that for every additional day a respondent adhered to foot-care regimen, they were 0.87 times less likely to have depression, controlling for all other factors in the model. The model was able to correctly classify 20.5% (sensitivity) of the respondents who had depression. The model was able to correctly identify 91.3% (specificity) of the respondents who did not have depression. Positive predictive value = 55.4%. Negative predictive value = 68.4%.

Direct Logistic Regression was performed to assess and identify the impact of factors on the likelihood that respondents would have Diabetes Distress. The model contained five independent variables (Days of Adherence to: General Diet, Specific diet, Exercise, Sugar testing and Foot-care). The full model containing all predictors was statistically significant, χ^2^ (5, N = 509) = 50.80, p < 0.001, indicating that the model was able to distinguish between respondents who had and did not have diabetes distress. The model as a whole explained between 9.7% (Cox and Snell R square) and 23.9% (Nagelkerke R squared) of the variance in distress status, and correctly classified 93% of cases. Only two of the independent variables made a unique statistically significant contribution to the model (Adherence to: Exercise and Foot-care). The strongest predictor of reporting distress was Foot-care, recording an odds ratio of 0.79. This indicated that the more days that a person adheres to Foot-care regime, the odds of him or her having diabetes distress decreases by a factor of 0.79, controlling for all other factors in the model. The odds ratio of 0.66 for Adherence to Exercise was also less than 1, indicating that for every additional day a respondent adhered to Exercise regimen, they were 0.66 times less likely to have distress, controlling for all other factors in the model. The model was able to correctly classify 5.6% (sensitivity) of the respondents who had distress. The model was able to correctly identify 99.8% (specificity) of the respondents who did not have distress. Positive predictive value = 66.67%. Negative predictive value = 93.16%.

A Hierarchical Multiple Regression was used to assess the ability of the five measures of SDSCA-General diet, Specific diet, Exercise, Blood glucose testing and Footcare to predict the diabetes distress after controlling for the influence of diastolic and systolic blood pressure and glycated hemoglobin (HbA1c). Preliminary analyses were conducted to ensure no violation of the assumptions of normality, linearity, multicollinearity, and homoscedasticity. systolic, diastolic blood pressure and HBA1c were entered in Step 1 explaining 6.3% of the variance in diabetes distress. After entry of the SDSCA variables at Step 2, the total variance explained by the model as a whole was 16.8%, F (8,500) = 12.60, p < 0.001. The control measures explain an additional 10% of the variance in DD, after controlling for blood pressure and glycated hemoglobin, R^2^ change = 10.4, F change (5,500) = 12.53, p < 0.001. In the final model glycated hemoglobin (β = 0.18, p < 0.001), Exercise (β = −0.16, p < 0.001) were statistically significant.

A Standard Multiple Regression was used to assess the ability of the five SDSCA measures (Specific diet, General diet, Exercise, Footcare and Blood glucose testing), to predict the depression scores. Preliminary analyses were conducted to ensure no violation of the assumptions of normality, linearity, multicollinearity, and homoscedasticity. The model which included the five measures of SDSCA explains 9.1% of the variance in depression with statistical significance (p < 0.001). Of the five variables, Exercise makes the largest unique contribution beta value (β = −0.14, p < 0.05), though General diet (β = 0.11, p = 0.03), and Footcare (β = −0.14, p < 0.05) also made statistically significant contribution. Specific Diet (β = −0.07, p = 0.12) and blood glucose testing (β = 0.06, p = 0.15) did not make a statistically significant contribution. In this model an increase in exercise scores by one standard deviation (SD = 2.18) the depression scores would decrease by 0.14 standard deviation units. Multiple Regression with five predictors R^2^ = 0.09, F (5,503) = 10.05, p < 0.001.

T-test were conducted to compare the adherence measures scores for participants with or without the clinical characteristics of hypertension, high cholesterol, kidney disease, eye disease, heart failure and stroke. There was a significant difference in diabetes self-care adherence specific diet scores for participants with heart failure (M = 4.04, SD = 1.60) and participants without heart failure (M = 4.60, SD = 1.64; t (507) = −2.20, p < 0.05 two tailed). The magnitude of the difference in the means (mean difference = −0.55, 95% CI: −1.04 to −0.60) was very small (eta squared = 0.009). There was no statistically significant difference in any of the other diabetes self-care adherence scores for participants reporting having or not the other clinical characteristics.

## 4. Discussion

There was a negative relationship between diabetes distress and all the parameters of self-care adherence, indicating that as the patients continued to practice self-care, the levels of diabetes distress decreased. This is an implication that adhering to self-care directives creates an enabling environment for diabetes distress levels to decrease. Additionally, there was also a negative relationship between the rates of depression and the patient's adherence to self-care routines. Moreover, the results indicated that the greater the number of days patients adhered to exercise and foot-care, the probability of having depression or diabetes distress decreased. This demonstrated that as the patients continued to strictly adhere to self-care guidelines, their levels of depression and diabetes distress reduced. These results are consistent with those obtained in previous studies [28],[29]. Endeavours targeting the reduction of barriers to effective diabetes management could possibly include considerations for the negative impact that diabetes distress and depression may have on self-care by screening for these affective disorders.

There was also a negative relationship between diastolic and systolic blood pressure, HbA1c and SDSCA general diet. This implied that the more the patients practiced adherence to general diet, the lower the blood pressure (towards normal blood pressure) as well as the percentage of HbA1c, this is consistent with previous studies that investigated the relevance of the adherence to dietary guidelines [30]. Evidence supports the significance of dietary patterns in the management of type 2 diabetes, as it influences a great number of cardiometabolic factors including glucose insulin homeostasis and blood pressure [31],[32]. Additionally, a positive relationship was observed between HbA1c and SDSCA blood glucose testing, an indication that increased adherence to blood glucose testing was directly related to an increase in HbA1c. This may be due to patients' consciousness to their sugar intake which may prompt them to test more and not simply to be more adherent to this regimen.

Contrary to other studies which found that generally older patients demonstrated poorer adherence [33], this study found that adherence to general diet and foot-care was greater in older participants. This may be as a result of the availability to the resource, time; a factor that may not be as readily available to younger persons with wider responsibilities. Older persons may no longer have the added pressures of work or dependent relatives, whereas younger persons may still have to manage their time around juggling such duties.

Non-adherence is often observed in patients as a behavioral problem, but it is more specifically, a multidimensional phenomenon established by an interaction of a number of diverse factors inclusive of demographic factors, condition or disease factors, psychological factors, health beliefs (knowledge and attitudes of patient), social factors, doctor-patient relationship, treatment regimen and settings [34].

While there exists a reasonably extensive amount of research in the area of psychological issues such as depression and its effects on patients' management, a small number of studies have examined how diabetes distress affects diabetes self-care management, and of these none were found to have concentrated on the unique context within the Caribbean. The findings of research targeted within this context therefore have the potential to inform interventions that can improve management and care in persons living with diabetes.

## 5. Limitations

This study however is not without limitations. Interpretations of the results must be considered within the context of the design; due to the cross-sectional design of the study, causal inferences cannot be examined, nor the impermanence of the effects. Future longitudinal work may be necessary to elucidate the probability of directionality and causality in the associations. The generalizability of the results is limited by first, the health-care setting of the study, (public health care clinics), and second the specific disease (type 2 diabetes).

## 6. Conclusions and recommendations

Affective disorder symptoms are present among Barbadian patients with type 2 diabetes mellitus, and are associated with poorer adherence to specifically, general diet and exercise. This evidence should expand the current understanding in recognizing that these emotional states have a significant influence on patients' diabetes self-care behavior and clinical outcomes. Additionally, the results suggest that as symptoms of affective disorders (DD, depression) increases, adherence to various self-care activities decreases. The overall severity of the affective symptoms could possibly be more important to self-care than whether or not an individual meets the criteria for the respective affective disorder. Any endeavors aimed at reducing barriers to effective diabetes management could benefit from the inclusion of screening for DD and depression, and an increase recognition that even sub-clinical symptoms of affective disorders may negatively impact diabetes care and management. The results emphasize the need for considerations that must be given to the individual mental well-being in the processes of management and care of T2DM.
